# Environmental Risk Assessment Using Neural Network in Liquefied Petroleum Gas Terminal

**DOI:** 10.3390/toxics11040348

**Published:** 2023-04-07

**Authors:** Lalit Rajaramji Gabhane, NagamalleswaraRao Kanidarapu

**Affiliations:** School of Chemical Engineering, Vellore Institute of Technology, Vellore 632014, India

**Keywords:** artificial neural network, consequence modelling, environmental risk, flammable vapour cloud, jet fire, vapor cloud explosion

## Abstract

The accidental release of toxic gases leads to fire, explosion, and acute toxicity, and may result in severe problems for people and the environment. The risk analysis of hazardous chemicals using consequence modelling is essential to improve the process reliability and safety of the liquefied petroleum gas (LPG) terminal. The previous researchers focused on single-mode failure for risk assessment. No study exists on LPG plant multimode risk analysis and threat zone prediction using machine learning. This study aims to evaluate the fire and explosion hazard potential of one of Asia’s biggest LPG terminals in India. Areal locations of hazardous atmospheres (ALOHA) software simulations are used to generate threat zones for the worst scenarios. The same dataset is used to develop the artificial neural network (ANN) prediction model. The threats of flammable vapour cloud, thermal radiations from fire, and overpressure blast waves are estimated in two different weather conditions. A total of 14 LPG leak scenarios involving a 19 kg capacity cylinder, 21 tons capacity tank truck, 600 tons capacity mounded bullet, and 1350 tons capacity Horton sphere in the terminal are considered. Amongst all scenarios, the catastrophic rupture of the Horton sphere of 1350 MT capacity presented the most significant risk to life safety. Thermal flux of 37.5 kW/ m^2^ from flames will damage nearby structures and equipment and spread fire by the domino effect. A novel soft computing technique called a threat and risk analysis-based ANN model has been developed to predict threat zone distances for LPG leaks. Based on the significance of incidents in the LPG terminal, 160 attributes were collected for the ANN modelling. The developed ANN model predicted the threat zone distance with an accuracy of R^2^ value being 0.9958, and MSE being 202.9061 in testing. These results are evident in the reliability of the proposed framework for safety distance prediction. The LPG plant authorities can adopt this model to assess the safety distance from the hazardous chemical explosion based on the prior forecasted atmosphere conditions from the weather department.

## 1. Introduction

Liquefied petroleum gas (LPG) is used as fuel in automobiles, industries, agriculture, and household contexts. Its high calorific value and low greenhouse gas emissions enhanced its domestic and industrial usage [1]. The rising demand for LPG can be fulfilled by establishing new plants. Since LPG occupies more volume at normal conditions and is more prone to fire and explosion hazards, it is stored in fully refrigerated conditions. Mechanical failures, weld failure, or rupture of LPG cylinders may lead to a loss of containment [2]. Small vapour leaks from LPG cylinders and rapid vaporization of tons of LPG are some of the hazards in LPG plants [3]. These hazards lead to risks such as boiling liquid expanding vapour cloud explosion, fireball, and jet fire [4]. The research on fire and explosion hazard analysis [5,6] and the lessons learned from the LPG accidents [7,8,9,10] revealed the need to address the LPG safety challenges [11,12]. In this context, quantitative risk analysis results are helpful in preparing emergency response plans [13,14]. 

Researchers performed risk and consequence analysis for the various units of process industries, such as storage tanks [14,15,16,17], dangerous chemicals [18], separators [19], and scrubbers [20]. In these studies, the numbers of scenarios considered are specific or limited. When a multimode of failures is considered, and to perform the consequence analysis, handling the data is a challenge for the risk management team. In this context, adopting the machine learning approach is a better decision [21,22]. Machine learning model implementation in process safety is a proven technique for consequence modelling [23], in forecasting failure modes in oil and gas pipes [24,25], and in predicting the boiling liquid expanding vapour explosions (BLEVEs) [26]. Decision-making is also an easy task through the implementation of artificial neural network models in risk assessment [27,28,29], in predicting the potentially hazardous chemicals in drinking water [30], and in forecasting the liquefied natural gas bunker prices [31]. 

In the literature, studies are available on risk or incident analysis of various sections of LPG bottling facilities [32]. To our best knowledge, there is no study on the risk analysis of multimode failure scenarios and the development of an ANN model to predict the threat zones. This research gap motivated the present study to conduct a fire and explosion analysis of the possible worst-case scenarios in one of Asia’s biggest LPG terminals. The worst-case scenarios simulation data are used to develop an ANN model to predict the threat zones related to the multimode failures in the LPG terminal. The ANN model proposed in this study is helpful in predicting the safety distances to be maintained in the LPG terminal for the weather forecasting input data from the Indian Meteorological Department (IMD). The results are also helpful for design engineers, disaster management authorities in making offsite emergency response policies, statutory industrial authorities in prescribing the mandatory safety distances between the facilities in the plant, and land use for land use planning around the plant, respectively.

## 2. Materials and Methods

**Figure 1:**
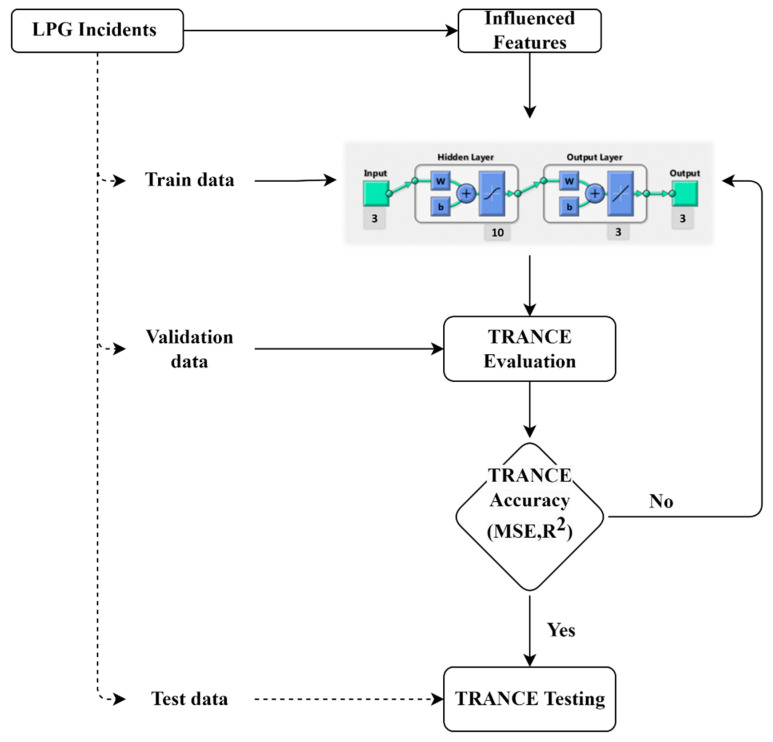
Systematic quantitative risk analysis of LPG plant using ANN methodology chart.

A risk assessment was carried out for an LPG bottling plant in an industrial area in western India. A process flow diagram of the plant, meteorological data of the local area, LPG chemical data, and ALOHA modelling software were used for the risk assessment, alongside a physical visit to the site. A three-step approach was adopted for the study, including the selection of a hazardous release event, simulation of hazardous release scenarios on an existing LPG plant, and analysis of the consequences in terms of impact threat zones. The systematic methodology suggested by the Centre for Chemical Process Safety [33] along with ANN is depicted in Figure 1. 

### 2.1. LPG Terminal

**Figure 2:**
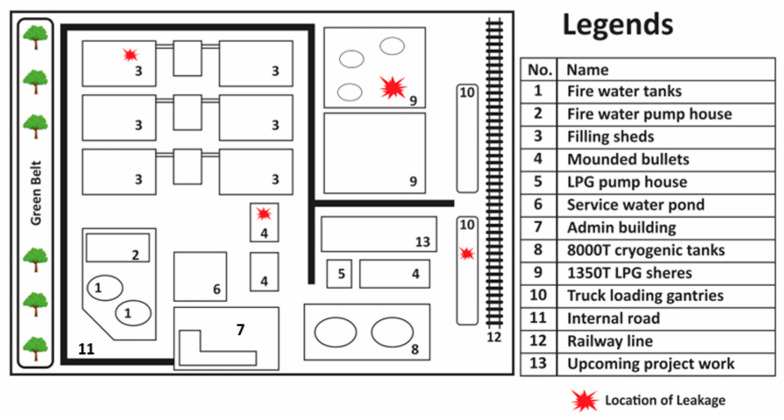
Block diagram of LPG terminal layout.

The selected LPG terminal is situated in an industrial area near western Maharashtra, India. The block diagram of the plant is shown in Figure 2. This LPG terminal is one of the largest in Asia. This terminal receives LPG from the refinery across the creek through the underwater pipeline and also by ships. The facility is located on about 160 acres of land. At the LPG terminal, 8 Horton spheres of 18 m diameter and 1350 MT capacity each, 2 cryogenic storage tanks of 8000 MT each, 2 mounded storage vessels of 600 MT each, and 3 mounded storage vessels of 900 MT each are present. The total LPG stored in the terminal is about 300,000 MT. The terminal supplies LPG to other nearby industries and bottling plants by tank trucks of 18 to 21 MT capacities, and by rail wagons. There is also a bottling facility; over 300 MT of LPG is bottled daily, with three carousels in operation. LPG is drawn from the mounded bullets for bottling in cylinders of various capacities (14.2, 19, and 45 kg) and is dispatched for domestic and industrial consumption. About 70 to 80 trucks loaded with filled LPG cylinders are dispatched daily from the plant. The current study covers the Horton spheres, tank trucks at the loading gantry, bottling operations, and associated facilities within the LPG terminal.

### 2.2. HAZID Methodology

Risk management in the oil and gas industry is vital in preventing accidents. In the oil and gas industry, risk assessment is carried out at the conceptual stage and until the end of the life cycle of the plant. Accidents in the oil and gas industry have catastrophic effects. Fire, explosion, and toxic gas releases from the oil and gas industry kill many human beings, damage assets, and impact the environment [34,35]. Over the last four decades, the management of risk from hazardous facilities’ operations is receiving increased attention. These major accidents emphasize the importance of process safety in the oil and gas industries. The oil and gas industry uses risk management to manage the threats and risks in their operation. Risk management has many steps. The first and key step of the risk assessment process is hazard identification. Various hazard identification techniques adopted in the oil and gas industry are as follows [36,37]: 1. HAZOP, 2. HAZAN, 3. Dow Fire and Explosion Index, 4. Mond index, 5. Safety audit.

**Figure 3:**
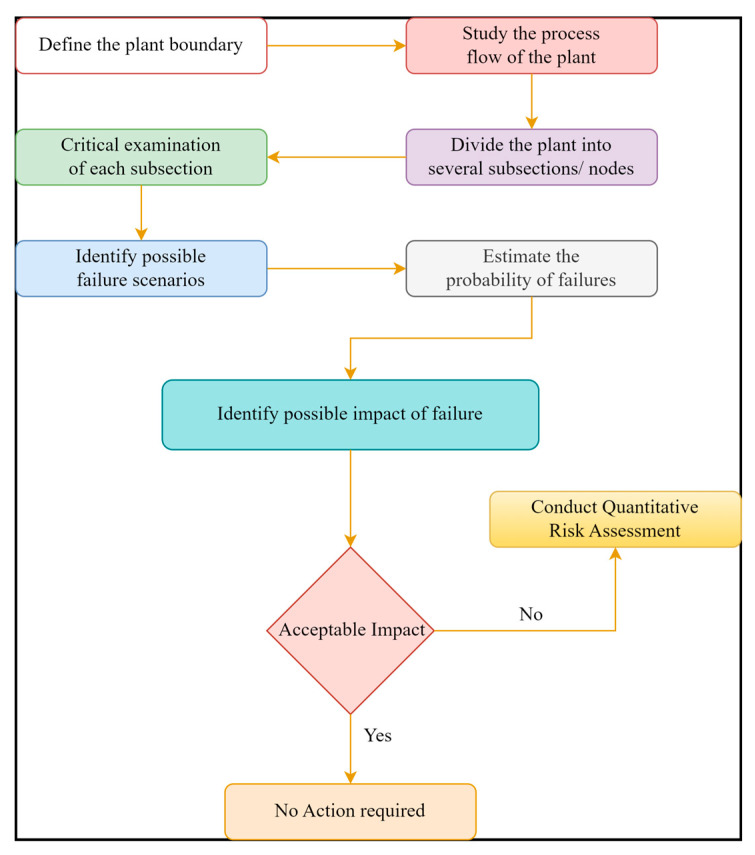
HAZID methodology flowsheet.

Hazard identification was made using the safety audit method. It is a semi-quantitative method to identify risk sources, causes, and consequences. Hazard identification identifies the hazards in the plant to be removed or managed. Usually, a multi-speciality team reviews the total project of the oil and gas plant and its effects on the safety, health and environment. The study follows a systematic methodology and uses a checklist to identify the various hazards and assess their influence on people and the environment. The steps followed for the hazard identifications are shown in Figure 3. This method needs to divide the process or plant into subsections and analyse these subsections according to harmful factors based on keywords to cover the inherent dangers, discover system problems, and define the risk level. A safety audit is conducted for the critical examination of each subsection for possible failures such as pipe leakage, valve leakage, ruptures, weld failures, other mechanical failures, etc., and to identify the potential impact of fire, explosion, and vapour cloud failures on humans, property, and the environment [36].

### 2.3. Hazard Identification and the Scenario Selection

A systematic hazard identification using the HAZID methodology was carried out. Necessary pieces of information on the LPG plant process, which includes geographical location through Google Maps, location meteorological data, site plan, P&IDs (process instrumentation diagrams), PFDs (process flow diagrams), operation procedures, physical and chemical specification of the material, etc., are collected.

The possible leak sources from the 19 kg capacity cylinder, 21 tons capacity tank truck, 600 tons capacity mounded bullet, and 1350 tons capacity Horton sphere are considered. Low-pressure LPG cylinders have domestic, commercial, and industrial uses. These cylinders come in various sizes in India, such as 4 kg, 5 kg, 6 kg, 10 kg, 12 kg, 12.5 kg, 14.2 kg, 15 kg, 17 kg, 19 kg, 21 kg, 33 kg, 47.5 kg, and 50 kg. This cylinder is manufactured per Indian standard I.S. 3196:2006 (Part I) [38]. The cylinders are made of 2.55 mm thick low-carbon steel, and the operating pressure in the cylinder is 1.66 MPa. An LPG cylinder of 19 kg capacity filled with LPG at a pressure of 1.66 MPa illustrates possible leak sources from its weld joints and the valve. Tank trucks of different capacities are used in India to transport the LPG gas from the gas terminals to the bottling plants and other valuable points of sale. In this study, a tank truck of 21 tons capacity, standing in the loading gantry, is considered for creating leakage scenarios. In India, various bullet sizes are manufactured, ranging from 5600 m^3^ to 20,000 m^3^. ASME (American Society of Mechanical Engineers) Sec VIII Div 2 and OISD-STD-150 are followed for the construction of bullets by manufacturers. ASME Sec VIII 129 Div 2 is a standard that deals with rules for constructing pressure vessels. OISD -STD-150 is a standard issued by Oil Industries Safety Directorate in India. It deals with the design and safety requirements of mounded LPG storage tanks.

The LPG bullet tank (mounded) of 600 MT capacity with 4.5 m diameter, 25 m length, and 22 mm thickness is considered for leakage scenarios. The operating pressure of this tank is up to 1.45 MPa. In India, various sizes of Horton spheres range from 10,000 mm to 22,000 mm in diameter. ASME sec viii Div I & II, PD 5500 standards [39] are used to construct the spherical tank. In addition, possible leak sources from a 1350 Tons LPG Horton Sphere of 72 mm shell thickness and having 1.71 MPa internal pressures are included in this study.

The range of leak sizes is representative of small and large leaks considered for the assessment based on the pipe sizes. All possible failure scenarios, including the likely near misses, were enumerated based on the knowledge of past events at LPG plants and engineering judgment. The events that did not occur, such as the failure of Horton sphere due to earthquake or aerial attacks, were assumed to carry out the risk assessment and estimate the consequences. The event tree of a pressurized flammable gas release and the individual risk acceptance criteria are the main factors in risk assessment [40].

In ALOHA (Areal Locations of Hazardous Atmospheres), the major chemical component propane is considered to model the hazardous effects of LPG. LPG discharge, dispersion, and thermal radiation effects and overpressures are simulated using ALOHA software as a function of process and atmospheric parameters. The meteorological data were based on the extreme day and night temperatures (highest daytime temperature in summer and lowest night-time temperature in winter at the location of the accident) in Mumbai, India, with the highest temperature in May 2022 and the lowest in December 2022. The relative humidity, wind directions, and wind speeds during the summer and winter are also inputs to ALOHA simulations. Rain is not considered since ALOHA does not provide rain as an input. A total of 14 worst-case scenarios given in Table 1 covering summer days and winter nights were selected for modelling, and the threats are quantitatively assessed.

### 2.4. ANN Topology

Machine learning techniques via ANNs, FL (fuzzy logic), ML (machine learning), P.R. (probabilistic reasoning), and E.A. (evolutionary algorithms) have been proven to exhibit incredible efficiency for problem-solving in a variety of applications, specifically in risk assessments to support decision-making processes in an economical and time-saving manner [41,42]. Among the traditional methods, ANN is one of the trustable alternative techniques to find and build a good relationship between process parameters. ANN has numerous applications such as system identification, process or equipment control, and risk and safety assessment [43]. The ANNs performance is an intellectual task similar to tasks performed by brain neurons, and it works by gathering, understanding, tuning, and storing knowledge during the training [44]. ANN is used in developing a noteworthiness surrogate model for a real-time process model [45]. Perceptron artificial nodes could correlate and create a new response for non-linear and complex process data. Multi-layered back propagation feed-forward neural network is preferable in chemical processes [46]. The proposed network was applied to predict the safety distance of the LPG dataset generated from ALOHA.

We examined the proposed framework capability with multi-failure scenarios over the single fault prediction completed earlier. The HAZID worst-case failure scenario data of the LPG terminal collected from NSCI (National Safety Council India) and the affected weather conditions were applied in ALOHA to generate safety distance measures. The effect of chemical explosion distance based on the weather conditions, air temperature, relative humidity, wind direction, wind speed, etc., were considered to measure the vicinity of hazardous chemicals. The chemical distances are classified as red, orange, and yellow zone, depending on chemical concentration and the severe risk to humans and/or the environment. One hundred and sixty (160) different weather conditions were applied, and the safety distances were measured. The noise and error in datasets were clustered, and unnecessary data were removed. Datasets were divided for training, validation, and testing purposes as 70%, 15%, and 15% of the total, respectively. The training data were applied to the ANN model to train the model and to establish a correlation between the input and target signals. The input layer ANN model has three features (air temperature, relative humidity, and wind speed) that are significant effects of a chemical explosion. The output layer of ANN has three targets, namely red, orange, and yellow threat zones for risk assessment. The validation datasets were used to evaluate the trained model, and the mandatory parameters were tuned to attain the high-performance accuracy of the ANN model. The complete computational work was carried out with the MATLAB neural network platform with 2022a version and 20 min of runtime on a core 5 computer and 8 GB RAM. The generated/collected data were trained and validated at a ratio of 70:15 using the developed network, and the measured error value was within the reasonable range.

The proposed ANN model’s performance depends on the number of hidden layers and hidden neurons in each layer based on activation and summation functions. The optimized ANN structure for threat and risk assessment is presented in Table 2. The collected data from the LPG terminal are pre-processed and applied to the ANN structure. The sigmoid activation function is used in the first hidden layer, and the linear activation function is used in the second hidden layer to generate a good relationship between input and output parameters. The Levenberg–Marquardt (L.M.) algorithm is used to train the learning process of the ANN model towards minimum error value and high accuracy. The L.M. algorithm repeatedly adjusts the weights and bias pattern of the ANN model with the back propagation feed-forward technique until the high-performance values are obtained. The Trainlm backpropagation algorithm works at high convergence speeds, even for large datasets. It will optimize the features iteratively until the predicted values reach an acceptable level. The specifications and overview of the proposed model information are provided in Table 2.

## 3. Results and Discussion

At the LPG terminal, LPG is stored in a 19 kg capacity LPG cylinder, 21 tons capacity tank truck, 600 MT mounded bullet, and 1350 tons capacity Horton sphere, respectively. The scenarios presented in Table 1 give information about the accidental release of LPG into the atmosphere. The released LPG evaporates immediately and causes harmful effects on people and the environment. The output results of the ALOHA simulation results are presented in terms of threat zones. In threat zones, the ground-level concentrations of LPG exceeded the level of concern (LOC). Here, the LOC indicates the threshold concentration of LPG that could harm the people and environment if exposed to a specific interval. LOCs are considered based on the AEGL (Acute Exposure Guideline levels) with a 1-hour minimum duration. In threat zones, the red zone is close to the release source, and LPG concentration will exceed 33,000 ppm within its area. The people within the red threat zone could experience life-threatening problems. Within the orange zone, the LPG concentration is considered as 17,000 ppm, and the people within the zone will suffer irreversible health effects and will not be able to escape the toxic chemical release. In the yellow threat zone, the concentration of the LPG is 5500 ppm. People in the yellow zone may suffer temporary symptoms such as irritation and discomfort. 

### 3.1. Modelling Flammable Area of Vapour Cloud

**Figure 4:**
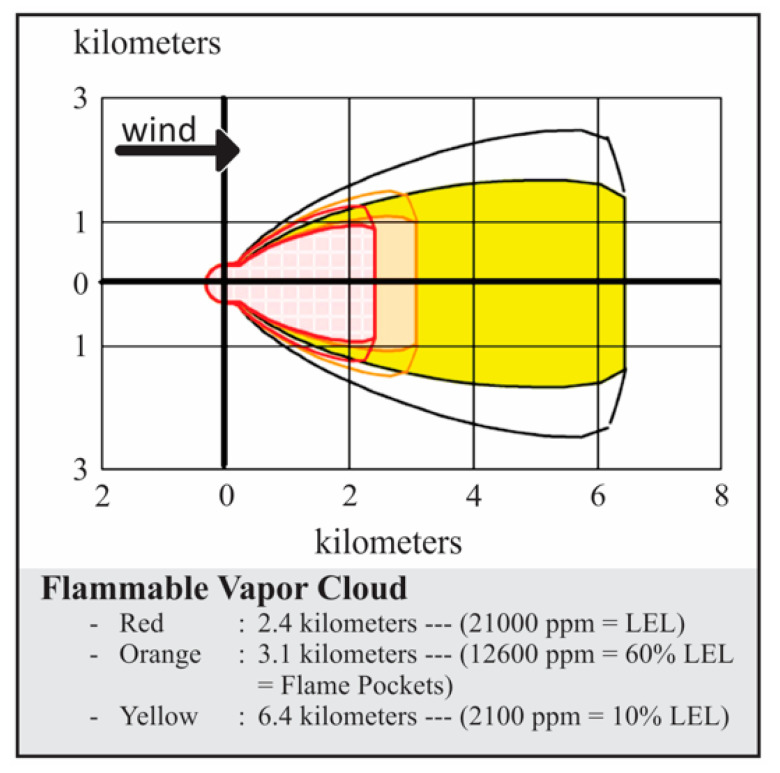
ALOHA simulation result for scenario S13 catastrophic rupture of Horton sphere.

The flammable vapour cloud area was modelled for all the worst-case scenarios in Table 1. Since the LPG is heavier than the air, the heavy gas model is used for the flammable area of the vapour cloud modelling purpose. Thermal radiations are computed using the solid flame model. From the simulation results, the catastrophic rupture of the Horton sphere is identified as the worst-case scenario. The amount of chemical mass released from the Horton sphere is 466,480 kg/h. The ALOHA simulation results of the catastrophic rupture of the LPG Horton sphere in summer daytime are given in Figure 4. Here, the hazards related to the fires resulting from the vapour cloud dispersion in the downwind direction, which contains a flammable chemical mixed with air, ignite and burn. The concentration profiles are indicated in red, orange, and yellow colours. The red zone with a lower explosive limit (LEL), the orange zone with 60% of LEL, and the yellow zone with 10% of LEL can be observed. The unignited vapour cloud will reach up to 2.4 km, building glasses may shatter up to a distance of 3.1 km, and the survival margin is 6.1 km. 

**Figure 5:**
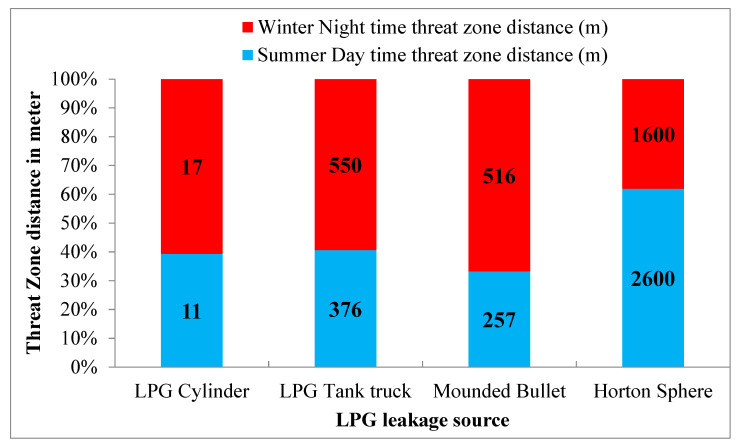
Flammable area of vapour cloud for lower explosive limit (LEL).

Modelling results for lower explosive limits (LEL) are depicted in Figure 5. From this, it is observed that the flammable vapour cloud spread to a distance of 17 m, 550 m, and 516 m for LPG cylinders, LPG tank trucks, and mounded bullets during the winter night. The Horton sphere spread up to 2600 m during summer daytime; this resulted in a flash fire in the case of ignition. It is observed that the damage is greater in the case of catastrophic rupture of the Horton sphere during summer daytime. The reason is that in the case of catastrophic rupture, chemicals are instantaneously released into the atmosphere, and the temperature is also high during the summer daytime.

### 3.2. Jet Fire Radiations

**Figure 6:**
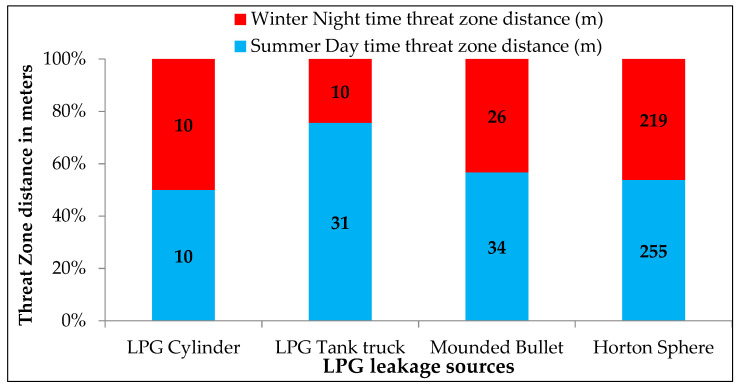
Jet fire radiation distances for lethal thermal radiation intensity of 37.5 kW/m^2^.

Jet fires result from the sustained release of LPG, immediate dispersion in the downwind direction, and ignition. A solid flame model is used to compute the jet fire radiations. Figure 6 shows the jet fire radiation distances for a lethal thermal radiation intensity of 37.5 kW/m^2^. Compared to the winter (night-time) threat distances, the summer thermal radiation distances are higher than the summer daytime thermal radiation distances. It indicates that the night-time scenarios cause less severe impact. The maximum radiation distance due to jet fire is 255 m from the Horton sphere. 

### 3.3. Fireball Radiations

**Figure 7:**
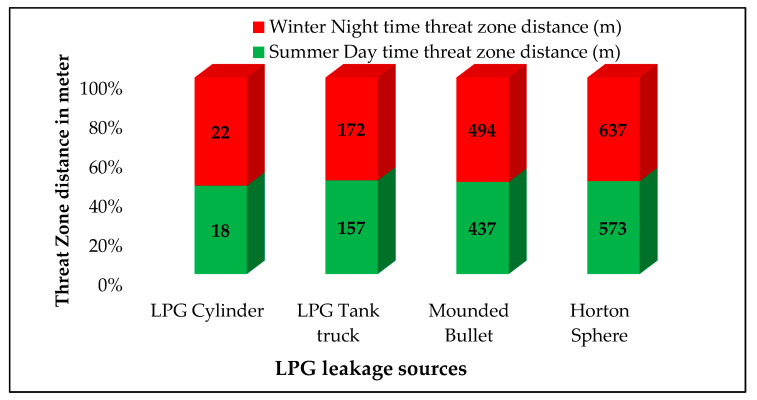
Fireball radiations of 37.5 kW/m^2^.

Fireballs occurred due to the explosion, pressurization, and immediate ignition of LPG gas in all the scenarios. The impact on people varies based on the duration of exposure and the radiation intensity. Thermal radiations are computed using the solid flame model in ALOHA. The radiations resulting from the LPG leakage from the LPG cylinder, LPG tank truck, mounded bullet, and Horton sphere for thermal radiation intensity of 37.5 kW/m^2^ are given in Figure 7. If the LPG that leaked from the LPG cylinder ignites, the resulting thermal radiation with 37.5 kW/m^2^ of radiant energy will reach up to 22 m. It will cause fatalities and an explosion of other cylinders. 

### 3.4. Blast Force

**Figure 8:**
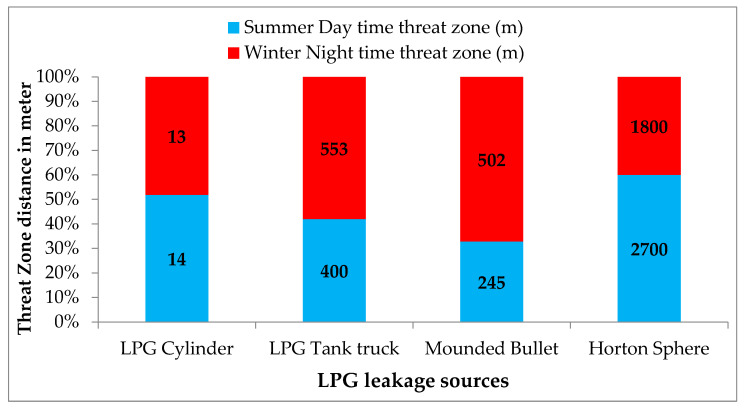
Blast force for 24.13 kPa pressure and 0.6 LEL.

The overpressure levels of concerns from ALOHA considered are 55.16 kPa, 24.13 kPa, and 6.9 kPa, respectively. The conditions are 55.16 kPa, destruction of buildings 24.13 kPa, serious injuries, and 6.9 kPa shattering of glasses. The scenario of blast force for the LPG cylinder, LPG tank truck, and mounded bullet during the winter night and summer daytime are depicted in Figure 8. The blast wave may cause severe injuries to people up to 2700 m away, and the building glasses may shatter at up to a distance of 3100 m for 10% of LEL. The severity of the blast force is high in the case of the Horton sphere, with a 0.15 m size leak in the summer daytime. 

### 3.5. Risk Assessment for Catastrophic Ruptures of LPG Horton Sphere

**Figure 9:**
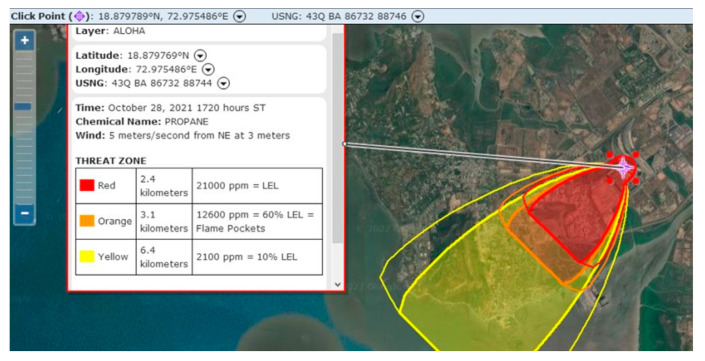
Risk mapping results of vapour cloud explosion for scenario S13 (catastrophic rupture of Horton sphere).

The simulation results of ALOHA for the hazardous effects of a catastrophic rupture of a 1350 tons LPG Horton sphere (scenario S13) mapped onto the Marplot are shown in Figure 9. The dangerous flammable vapour cloud reaches the nearby residential township. A leakage unignited vapour cloud will reach up to 2400 m in the downwind direction. The results of risk mapping from Figure 8 can be used for emergency planning and training. 

In the case of immediate ignition of the hazardous material at the source, the thermal radiations of 37.5 kw/m^2^ intensity from BLEVE (boiling liquid expanding vapour explosion) will reach up to 563 m. They may cause significant property damage and fatalities. In the case of a vapour cloud explosion, a 24.13 kPa blast wave may cause severe human injuries up to 2.6 km and building glasses up to 3 km distance may shatter. Persons within 1.9 km will receive second-degree burn injuries as they will be exposed to thermal radiation for over 20 seconds. The firefighters will also receive burn injuries if they are not wearing heat-resistant protective clothing. 

### 3.6. Performance of ANN Model

The 160 threat zone safety distances were collected from different weather conditions for the ANN model. The 160 datasets are split into 112, 24, and 24 for training, validation, and testing for the ANN model. The training data are applied to the ANN model to train the model and to establish a correlation between the input and target signals. 

**Figure 10:**
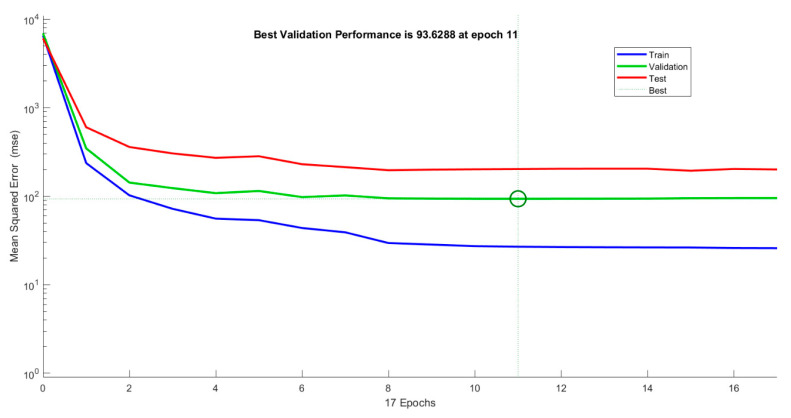
Leaning rate curve for ANN model performance with training, validation, and testing datasets in LPG plant.

The ANN model is iterated multiple times to reach the minimum error value. The learning rate of the proposed ANN model is filmed in Figure 10. A smaller vertical distance or error distance between blue and green lines represents excellent performance of the ANN model. 

Overfitting of the ANN model occurs in the case of the error being too small, and underfitting in the case of the error value being too large in training, validation, and testing datasets [47].

These curves show the minimum error of 93.6288 at epoch 11 of total epoch 17. There is no over- or underfitting of the ANN model found from the curves of the learning rate. Thus, the ANN model for threat and risk assessment was established successfully. The performance of the ANN model can also be evaluated from important parameters called the coefficient of determination (R^2^) and mean square error (*MSE*).

The mathematical expressions for *MSE* and R^2^ are represented as follows [48]:

(1) $$MSE=\frac{1}{N}\sum_{i=1}^{N}{{(y}_{ANN,i}-y_{exp,i})}^{2}$$

(2) $$R^{2}=1-\frac{\sum_{i=1}^{N}{(y}_{ANN,i}-y_{exp,i})}{\sum_{i=1}^{N}{(y}_{ANN,i}-y_{m})}$$

Here, $y_{ANN,i}$ represents *ANN* prediction values from the proposed model, $y_{exp}$ is true experimental values or target features, and $y_{m}$ is the mean of target values.

**Figure 11:**
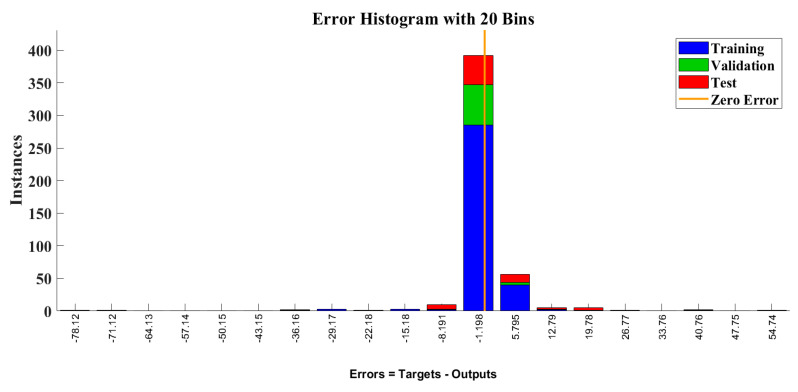
Error histogram plot of the LPG plant training, validation, and testing datasets.

The error value from the ANN model and the target is mentioned in Figure 11: the histogram graph of training, validation, and testing are plotted with a zero line at 20 bins. The error values of testing, validation, and training displayed a linear increment and decrement order change. The error value of most datasets is near zero, which indicates a muscular prediction strength of the ANN model for the chemical explosion.

**Figure 12:**
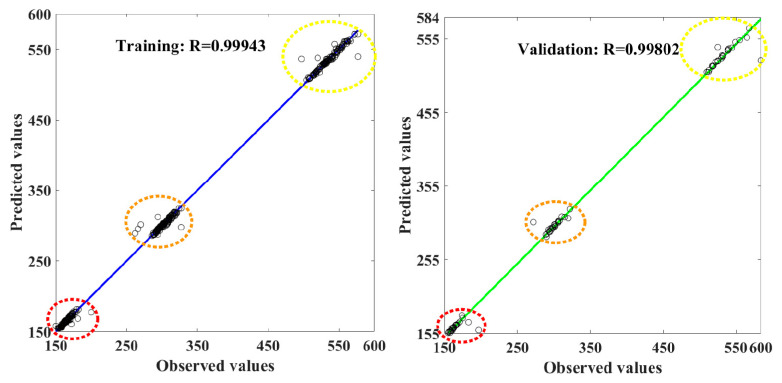
The correlation coefficient for three threat zone prediction values from the ANN model in training and validation in the LPG plant.

A new dataset (separated for testing) was used to predict the threat zone perimeter and distance using developed ANN, and the responses were gathered. The ANN error values from training, validation, and testing are obtained, and the same is tabulated in Table 3. The ANN-predicted values of training and validation were compared with observed or original datasets, and the regression plots were drawn in Figure 12. The regression value of ANN values coefficient of correlation is 0.9994 and 0.998 in training and validation, respectively.

The mean absolute error value of ANN prediction was 26.9212 and 9306288 in training and validation, respectively. In testing, prediction values of the ANN model from the new data sets were analysed; the R^2^ is 0.9958, and the MSE value is 202.9061. The overall prediction from all phases has an R^2^ of 0.99866. 

**Figure 13:**
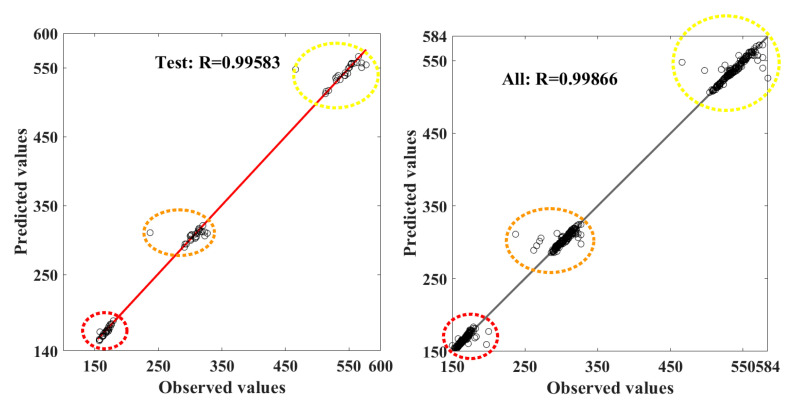
Correlation coefficient for three threat zone prediction values from ANN model in testing and all together in LPG plant.

The same test and overall prediction are plotted in Figure 13. The chemical explosion’s red, orange, and yellow regions are highlighted in the same way. Past research states that this range of values for threat and risk assessment in LPG plants is acceptable. 

### 3.7. Limitations of the Study and ANN Model

The study was limited to estimating the consequences of hazardous releases during two extreme weather conditions (i.e., extreme summer and extreme winter); hence, risk mitigation measures were not studied and not recommended. Industrial data from the LPG terminal complex in western India and risk mitigation measures were not studied or recommended. The failure scenarios data were used as input for the ALOHA. The ANN model dataset was generated based on the LPG’s weather conditions and failure scenarios together. In this context, authors can generate datasets by changing weather conditions in ALOHA based on their plant location. These same data can be applied to this ANN model, but this proposed accuracy may vary. This model can change or modify the hyperparameters such as training algorithm, number of neurons, layer size, epoch size, and dataset splitting to obtain accurate predictions from the model.

## 4. Conclusions

In this research, a fire and explosion hazard assessment of an LPG cylinder of 19 kg capacity, LPG tank truck of 21 tons capacity, mounded bullet of 600 tons capacity, and Horton Sphere of 1350 tons capacity at one of the biggest Asian liquefied petroleum gas terminals located in India was conducted. Simulations were performed using ALOHA for daytime in summer and night-time in winter. The threat zones for flammable vapour clouds, thermal radiations, and blast force were plotted. The plots showed that the threat zones extended to a larger area or longer distances during night-time in most cases compared to the daytime. In the case of a major disaster involving a catastrophic rupture of the Horton sphere or other large vessels, the plant firefighting facilities will be of very limited use, requiring the deployment of a public disaster management team. The lowest minimum safety distance of 3 m and highest minimum safety distance of 30 m, as prescribed in regulatory requirements, i.e., Static & Mobile Pressure Vessel (Unfired) Rules 2016, are grossly inadequate. In the case of disaster, the damage will be much beyond the regulatory safety distances. From this point of view, the proactive online monitoring risk assessment of LPG terminals is of utmost importance. We examined the proposed framework capability with multi-failure scenarios over the single fault prediction performed earlier. 

The suggested ANN framework model has been validated with a high accuracy of 99.86% to predict the distance travelled by the LPG leaks. The prediction results show that the coefficient of determination values in training, validation, and testing are 0.9994, 0.998, and 0.9958, respectively. The mean absolute error values of the ANN model in threat and risk zone prediction are 26.9212, 93.6288, and 202.906.

These highly accurate results are evident in the reliability of the proposed framework for safety distance prediction. This model can be adopted for LPG management to assess the safety distance from the hazardous chemical explosion based on the prior forecasted atmosphere conditions from the weather department.

This work provides valuable insights that in the case of a catastrophic failure of the LPG Sphere, the human population, flora and fauna, and property located within 1.8 km of the LPG Sphere will be affected most. Local disaster management authorities should consider the probabilities of such LPG disasters and equip themselves to tackle emergencies, such as by ensuring they have necessary firefighting equipment, trained personnel, emergency transportation facilities, and medical facilities. The study also provides useful data for plant designers, land use planners, and regulatory authorities to determine the safety distances within and around such plants. The findings will also be helpful to other researchers working in disaster management. 

## Figures and Tables

**Table 1:** The list of worst-case scenarios considered for the LPG terminal.

Scenario Name	Scenario Description	Leak Sizes in m
S1	A leak from 19 kg capacity LPG cylinder in filling shed in summer daytime (5D condition)	0.02
S2	A leak from 19 kg capacity LPG cylinder in filling shed in winter night (2F condition)	0.02
S3	Leak from 21 Tons capacity LPG Tank Truck in Loading gantry in summer daytime (5D condition)	Full bore rupture (0.15)
S4	A leak from 21 Tons capacity LPG Tank Truck in Loading gantry in wintertime (2F condition)	Full bore rupture (0.15)
S5	A leak from 21 Tons capacity LPG Tank Truck in Loading gantry in summer Day time (5D condition)	0.1
S6	Leak from 21 Tons capacity LPG Tank Truck in Loading gantry in wintertime (2F condition)	0.1
S7	A leak at 600 MT Mounded LPG Bullet in summer daytime (5D condition)	0.1
S8	A leak at 600 MT Mounded LPG Bullet in winter night-time (2F condition)	0.1
S9	A leak from 1350 Tons capacity LPG Horton Sphere in summer daytime (5D condition)	Full bore rupture (0.15)
S10	A leak from 1350 Tons capacity LPG Horton Sphere in winter night-time (2F condition)	Full bore rupture (0.15)
S11	A leak from 1350 Tons capacity LPG Horton Sphere in summer daytime (5D condition)	0.1
S12	A leak from 1350 Tons capacity LPG Horton Sphere in winter night-time (2F condition)	0.1
S13	Catastrophic rupture of 1350 Tons LPG Horton Sphere of 1350 MT in summer daytime (5D)	Catastrophic rupture
S14	Catastrophic rupture of 1350 Tons LPG Horton Sphere of 1350 MT in winter night-time (2F)	Catastrophic rupture

**Table 2:** Overview of Proposed ANN model.

Particulars	Specifications
Number of neurons	10 (First layer), 3 (Second layer)
Number of features in input and output	3, 3
Training algorithm	Feed-Forward Backpropagation
Optimization algorithm	Trainlm (Levenberg–Marquardt)
Activation function	Sigmoid (hidden layer), Linear (output layer)
Performance evaluation index	MSE, R^2^
Number of epochs	17
Number of attributes	160

**Table 3:** Performance of developed ANN model for the chemical explosion.

	Observations	MSE	R^2^
Training	113	26.9212	0.9994
Validation	24	93.6288	0.998
Testing	24	202.9061	0.9958

## Data Availability

The data used in this work are incorporated into the paper itself.
